# The Microtubule End Binding Protein Mal3 Is Essential for the Dynamic Assembly of Microtubules during *Magnaporthe oryzae* Growth and Pathogenesis

**DOI:** 10.3390/ijms25052672

**Published:** 2024-02-26

**Authors:** Ningning Shen, Libo Han, Zixuan Liu, Xianya Deng, Shuai Zhu, Chengyu Liu, Dingzhong Tang, Yuanbao Li

**Affiliations:** 1 State Key Laboratory of Ecological Control of Fujian-Taiwan Crop Pests, Key Laboratory of Ministry of Education for Genetics, Breeding and Multiple Utilization of Crops, Plant Immunity Center, Fujian Agriculture and Forestry University, Fuzhou 350002, China; shenningning1994@163.com (N.S.); hanlibo@im.ac.cn (L.H.); liuzixuan220221@163.com (Z.L.); dengxianya666@163.com (X.D.); shuaiz22620@163.com (S.Z.); liuchengyufafu@163.com (C.L.); 2 College of Agriculture, Fujian Agriculture and Forestry University, Fuzhou 350002, China; 3 School of Future Technology, Fujian Agriculture and Forestry University, Fuzhou 350002, China

**Keywords:** rice blast, *Magnaporthe oryzae*, infection mechanism, microtubule cytoskeleton, dynamic assembly, nucleus division, microtubule plus end

## Abstract

Cytoskeletal microtubules (MTs) play crucial roles in many aspects of life processes in eukaryotic organisms. They dynamically assemble physiologically important MT arrays under different cell conditions. Currently, aspects of MT assembly underlying the development and pathogenesis of the model plant pathogenic fungus *Magnaporthe oryzae* (*M. oryzae*) are unclear. In this study, we characterized the MT plus end binding protein MoMal3 in *M. oryzae*. We found that knockout of *MoMal3* results in defects in hyphal polar growth, appressorium-mediated host penetration and nucleus division. Using high-resolution live-cell imaging, we further found that the *MoMal3* mutant assembled a rigid MT in parallel with the MT during hyphal polar growth, the cage-like network in the appressorium and the stick-like spindle in nuclear division. These aberrant MT organization patterns in the *MoMal3* mutant impaired actin-based cell growth and host infection. Taken together, these findings showed that *M. oryzae* relies on MoMal3 to assemble elaborate MT arrays for growth and infection. The results also revealed the assembly mode of MTs in *M. oryzae*, indicating that MTs are pivotal for *M. oryzae* growth and host infection and may be new targets for devastating fungus control.

## 1. Introduction

Rice is one of the most important crops worldwide, as it feeds more than half of the world’s population. The fungal pathogen *Magnaporthe oryzae* (*M. oryzae*) seriously threatens rice production. Rice blast may cause severe disease, which can result in massive losses in plant mass and yield [1,2]. The pathogen successfully evolves a set of strategies for infecting host plants. After the fungus lands and adheres to the rice leaf surface, *M. oryzae* continuously changes hyphal morphology to construct integrated infection structures, such as the appressorium, the infection peg and the secretion apparatus, to invade and proliferate inside host cells [3,4,5]. Understanding the molecular and cellular mechanism of how these elaborate infection structures are formed is crucial for elucidating the pathogenesis of this devastating fungus.

Two major classes of the cytoskeletal network are conserved in higher eukaryotes. The first is the actin cytoskeleton, which is formed by the polymerization of globular actin into filamentous actin; the second is the microtubule (MT), which is composed of α- and β-tubulin heterodimers. Both types of cytoskeletal networks require the functions of corresponding binding proteins to regulate their active polymerization or depolymerization [6]. Through rapid polymerization and depolymerization, microfilaments (MFs) and MTs build a highly specific and dynamic framework during various physiological processes. The assembly and functions of the actin cytoskeleton have been elucidated for the plant disease fungus *M. oryzae*. A dense actin network composed of actin patches and actin cables connected to the spitzenkörper (Spk) was dynamically synthesized at the apex of hyphal cells during *M. oryzae* polar growth [7]. In the appressorium, a toroidal F-actin network is formed for pressure sensing and generation, which facilitates the formation of penetration pegs to breach the leaf surface [8,9]. Thus, proteins that regulate actin assembly, such as MoFim1, MoCAP, MoEND3, septins and Pmk1, are crucial for *M. oryzae* development and infection [8,10,11,12,13]. The septin-mediated actin ring organization within the appressorium was designed as a target for devastating fungus control [14].

The principles and dynamic regulatory mechanism of the MT assembly array in *M. oryzae* are currently unclear. It was previously indicated that they were assembled as vertically orientated polarized microtubule arrays, with their plus ends toward the appressorium pore [15]. However, additional details were needed for an in-depth analysis. Some clues from other filamentous fungi indicate that the MT could also organize some ordered networks and are required for development or pathogenesis. In the filamentous fungus *Aspergillus nidulans* (*A. nidulans*), MTs initiate polymerization at the spindle pole body (SPB), where the MT minus ends are often fixed [16]. In the cell tips of *A. nidulans* and *Neurospora crassa* (*N. crassa*), most cytoplasmic MTs are organized to orient their dynamic plus ends toward the hyphal tip [17,18,19]. Knockout of tubulin or MT-associated proteins (MAPs) affects fungal growth and disease progression. *Fusarium graminearum* (*F. graminearum*) α1 and β2 tubulin were shown to be indispensable for deoxynivalenol (DON) production [20]. The MT-end-binding protein FgEB1 in *F. graminearum* and EBA in *A. nidulans* were shown to regulate the polar growth of the fungus [21,22]. All these reports provide clues about the crucial functions of MTs in fungi. However, revealing the mechanism of MT organization and assembly in the model plant disease fungus *M. oryzae* is urgently needed.

In eukaryotic cells, MTs are usually organized as cortical transverse arrays and special MT structures in dividing cells, such as the preprophase band (PPB), phragmoplast arrays in plant cells and the bipolar spindle array [23]. The dynamic assembly of MTs, the relevance of microtubules and the formation of cellular structures are spatially and temporally controlled by numerous microtubule-associated proteins (MAPs) and molecular motors [24]. γ-tubulin, which is homologous to α- and β-tubulins, can form a protein complex and subsequently serve as an initiation factor for MT-nucleating factors in eukaryotes [25,26]. After initiation, the microtubule plus-end-tracking proteins (+TIPs) respond to the extension of the microtubule in several ways: (1) the newly formed MT assembles parallel to the existing MT, called MT-dependent MT nucleation; (2) the newly formed MT assumes 40° to the extant MT, which is called branched MT nucleation; and (3) the newly formed MT can also form a de novo MT in the presence of the γ-tubulin protein complex and +TIPs, which is called de novo MT nucleation [23]. +TIPs are structurally and functionally diverse microtubule regulators, for example, the end-binding protein 1 (EB1), XMAP215, some kinesins and dynein proteins. They are distinguished by their ability to concentrate at growing microtubule ends [24].

Microtubules play fundamental roles in many essential biological processes, but the assembly principle, organization and functions of these microtubules have not been clearly elucidated in the rice fungal disease rice blast. In this study, we investigated MT assembly in the model pathogenic fungus *M. oryzae* and performed high-resolution live-cell imaging to reveal the architecture and dynamics of MTs in *M. oryzae*. We demonstrated that MT organizes different and unique arrays involved in hyphal polar growth, appressorium formation and mitosis. Furthermore, we showed that the *M. oryzae* MT end binding protein (MoMal3) serves as an important factor for MT dynamic polymerization. Knockout of *MoMal3* results in apparent defects in MT dynamics and organization, which severely decreases fungal infection ability. Our results provide evidence that the development and elaboration of subtle MT arrays in *M. oryzae* is a pivotal factor for fungal development and pathogenesis. Moreover, this study may lead to the development of an exciting research field with the potential to open new avenues in which the microtubule cytoskeleton is a target for antifungal treatments.

## 2. Results

### 2.1. MoMal3 Is Required for M. oryzae Development and Host Infection

**Figure 1:**
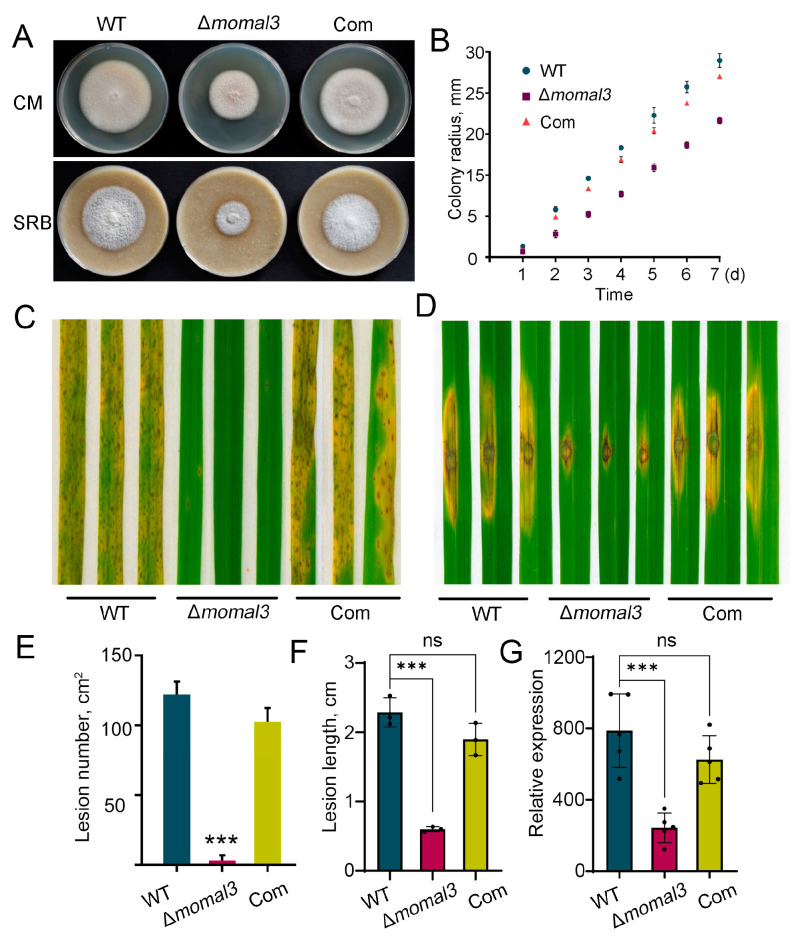
Growth and plant infection defects of the *MoMal3* mutant. (**A**) Seven-day-old cultures of the WT, Δ*momal3*, and complemented strain (Com) strains on CM and SRB media. (**B**) The graphs indicate the hyphal growth rate of the WT, Δ*momal3* and complemented strains. The colony diameters on CM plates were measured 7 days after inoculation. The error bars indicate the SDs calculated for three replicates. (**C**,**D**) Pathogenicity assay of the WT, Δ*momal3*, and complemented strains via whole-plant spray (**C**) and punch inoculation (**D**) assays. Conidia (10^5^ spores/mL) from the WT, Δ*momal3* and complemented strains were sprayed onto or removed from the punctured rice leaves (*O. sativa* cv. *Nipponbare*). Photographs were taken 6 days after infection. (**E**,**F**) Quantification of the lesion number per cm^2^ (**E**) and lesion area (**F**) of the rice leaves from the rice leaves shown in (**C**,**D**). The error bars represent SDs (*n* = 20), and the asterisks (***) represent significant differences (*p* < 0.001), ns: no significant difference. (**G**) Relative fungal counts of the *M. oryzae Pot2* gene compared to those of the rice *ubiquitin* gene determined by qPCR. The data represent the mean and standard deviation of three biological replicates. Three technical replicates were performed for each biological sample. Asterisks represent significant differences (*** *p* < 0.001, Student’s *t* test), ns: no significant difference.

Mal3 is homologous to the microtubule end binding 1 (EB1), which has been shown to be involved in MT integrity in eukaryotes [21,27,28]. In the sequenced *M. oryzae* strain Y34, one *Mal3* gene was encoded in the genome [29]. We then knocked out this gene in Y34 using a one-step gene replacement strategy (Figure S1). We also constructed a complemented strain by expressing *MoMal3* driven by its native promoter in the obtained mutant (termed Com). We then tested whether the *MoMal3* mutation causes defects in *M. oryzae* and found that the mutant strains formed colonies of reduced size in both complete medium (CM) and straw rice bran (SRB) medium (Figure 1A,B). Next, we analyzed the infection ability of the Δ*momal3* strain. The same number of conidia was used for spray or punch inoculation assays for rice leaf infection. The results showed that the virulence of the Δ*momal3* strain was significantly lower than that of the WT and complemented strains. In particular, in the spray-inoculation assay, the infection ability of Δ*momal3* was almost completely abolished (Figure 1C–G).

Notably, some conidia of Δ*momal3* did not exhibit the typical three-cell morphology, indicating that confused cell division may exist in the mutant. We then labelled the nuclei of the WT, Δ*momal3* and complemented strains by expression of the nuclear localization signal (NLS)-mCherry fusion protein. Although the overwhelming majority of the WT and complemented conidia contained three cells, those in the Δ*momal3* strain had various numbers of nuclei (Figure S3A–G). One to five nuclei were present in the population conidia of Δ*momal3* (Figure S3H). Next, we investigated whether the polar growth of Δ*momal3* was affected. Through live-cell imaging, we found that the growth rate of the Δ*momal3* hyphae was significantly decreased (Figure S3I; Supplemental Movie S1). Normally, *M. oryzae* hyphal tip cells exhibit sustained unidirectional growth, such that the hyphae exhibit linear and straight appearances. However, the Δ*momal3* tip cells exhibited wavy or curved growth patterns compared with those of the WT and complemented strains (Supplemental Movie S1 and Figure S3J–L).

In addition, we found that appressorium formation was affected in the Δ*momal3* strain when the conidia were cultured on hydrophobic glass (Figure S3M). The incipient collapse assay showed that the rate of collapse of the appressoria of the Δ*momal3* strain was significantly greater than that of the WT and complemented strains (Figure S3N), suggesting that MoMal3 may contribute to appressorial turgor generation.

Taken together, these findings showed that MoMal3 is crucial for *M. oryzae* development and pathogenesis.

### 2.2. MoMal3 Is an MT Plus (+tip) End-Binding Protein

**Figure 2:**
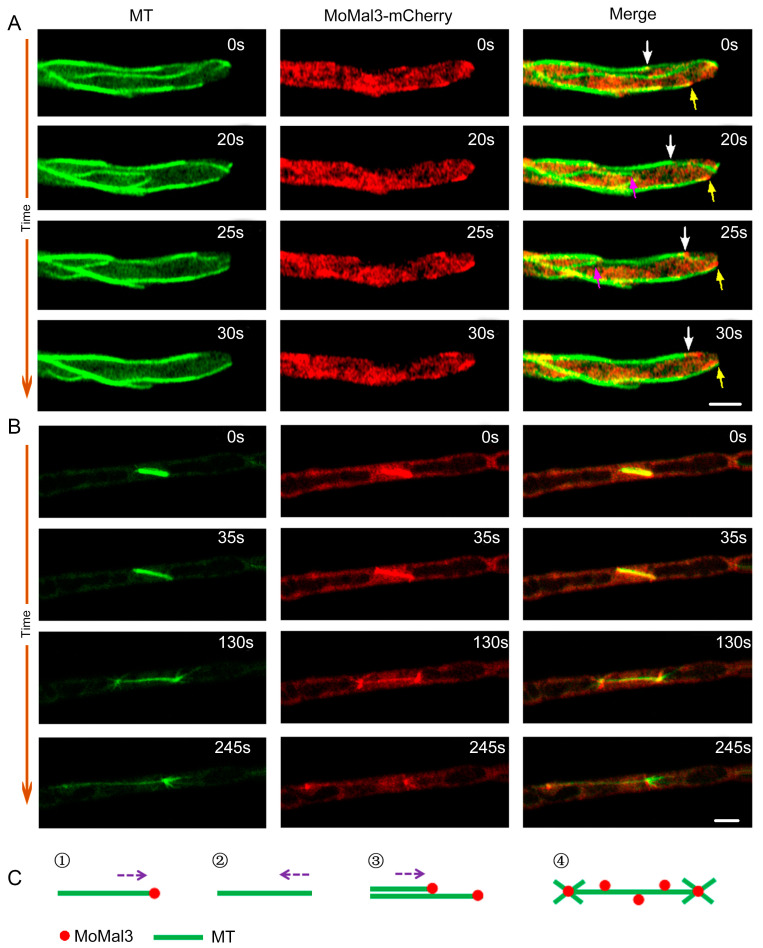
Localization of MoMal3 in *M. oryzae.* (**A**) Expression of *p*MoMal3-MoMal3-mCherry in β-tubulin-GFP-labelled Δ*momal3* vegetative hyphae. The numbers at the top right corner indicate the timestamps (S). The white and yellow arrows indicate that MoMal3 guides +tip MT polymerization along an existing MT to form MT bundles and typical single MT +tip elongation, respectively. The purple arrow indicates that MoMal3-mCherry does not localize to the depolymerized MT+ tip. The corresponding movie is provided as Supplemental Movie S2. Bars = 5 μm. (**B**) MoMal3-mCherry localized to a developing MT spindle. The corresponding movie is provided as Supplemental Movie S3. Bars = 5 μm. (**C**) Subcellular localization pattern of MoMal3 in *M. oryzae* shown in (**A**,**B**). ①–④ indicates the four MoMal3 localization patterns. And the purple arrows indicate the growing direction of MT.

Considering that MoMal3 is an MT-associated protein, we observed the localization of MoMal3 in *M. oryzae*. MoMal3-mCherry driven by its native promoter and a β-tubule-GFP were coexpressed with Δ*momal3*, and the colocalization of GFP and mCherry was observed via high-resolution live-cell imaging microscopy. Time-lapse imaging of the vegetative hyphae revealed that during the polymerization of MT, MoMal3 was localized mainly at the growing +tip end of the MT (Supplemental Movie S2). These included MoMal3-mCherry localized at the +tip of a growing single MT (Figure 2A, yellow arrows) or an MT parallel to an existing MT (Figure 2A, white arrows) to form MT bundles. We also noticed that MoMal3 did not localize to the +tip MT end when the MT was shrinking (Figure 2A, purple arrows).

Fluorescence microscopy also revealed that MoMal3-mCherry localized to the MT spindle during fungal mitosis (Figure 2B; Supplemental Movie S3). A schematic diagram was constructed to show the localization patterns of MoMal3 according to the observations (Figure 2C).

### 2.3. Knockout of MoMal3 Results in Defects in MT Dynamic Assembly during Hyphal Polar Growth

**Figure 3:**
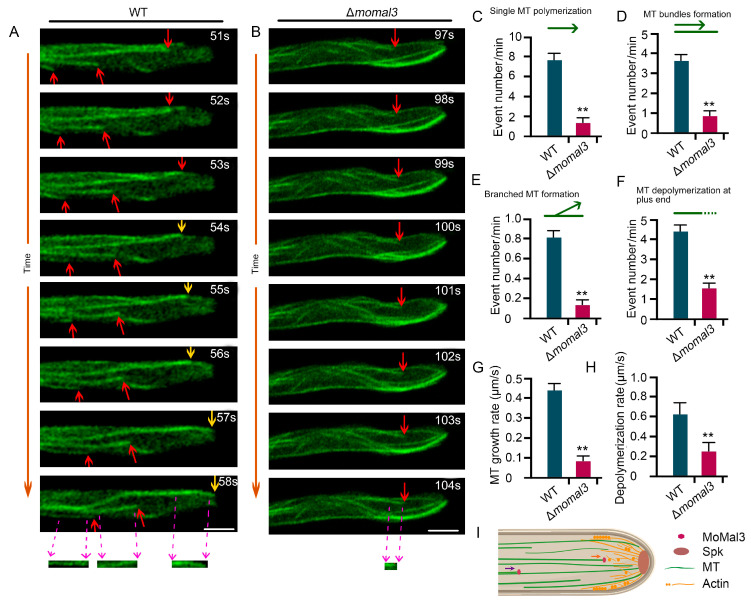
Microtubule organization in the WT and Δ*momal3* hyphae. (**A**,**B**) Representative time-lapse images showing the MT dynamics in growing WT (**A**) and Δ*momal3* (**B**) hyphae. Thirty cells were observed for both the WT and Δ*momal3* strains, and similar observations were made. The corresponding movie is provided as Supplemental Movies S4 and S5. The red arrows indicate single MT elongation events, and the yellow arrows indicate a growing MT along an existing MT bundle to form an MT bundle. The purple dashed arrows indicate the elongated MT at the same time period. Bars = 5 μm. (**C**–**H**) Statistical analysis of the number of single MTs: (**C**) MT bundle formation; (**D**) branched MT formation; (**E**) MT depolymerization at the +tip end; (**F**) single MT elongation rate; (**G**) and single MT depolymerization rate in the growing hyphae of the WT and Δ*momal3* strains, provided in Supplemental Movies S4 and S5. The green arrows indicate the MT assembly direction and pattern. And the dash line in (F) indicates the depolymerization of MT at the MT (+) plus end. The error bars represent SDs (*n* = 50), and the asterisks (**) represent significant differences (*p* < 0.01). (**I**) A proposed model showing the organization of the MT at the hyphal tip. Arrows indicate the MT assembly direction.

Next, we investigated the organization and dynamic assembly of MT in *M. oryzae* hyphae, which have not been studied previously. We expressed β-tubule-GFP in both the WT and Δ*momal3* strains. High-resolution live-cell imaging revealed that in the WT hyphae, MTs were assembled as fine filaments parallel to the hyphal growth axis. Along with hyphal growth, dynamic MT polymerization occurred at the +tip end, where new single MTs or bundled MTs were formed toward the hyphal tip (Figure 3A). We also attempted to introduce an augmin protein (ELQ39527) fusion with mCherry into WT and Δ*momal3* strains to clearly monitor the polymerization parameters of MT. Unfortunately, we failed to detect augmin-mCherry fluorescence. We speculated that this difference may be attributed to the weak expression of this gene driven by the native promoter of this augmin. Thus, we next directly calculated the MT assembly parameters by evaluating the occurrence of MT events. We found that single MT elongation, MT bundle formation and branched MT formation occurred 7.4, 3.4 and 0.8 times per minute on average, respectively. The average growth rate of the MT at the +tip end is approximately 0.44 µm/s. In addition, MT + tip depolymerization could be easily observed in the growing hyphae and occurred 4.2 times per minute on average (Figure 3; Supplemental Movie S4). We did not observe typical MT severing, MT crossover formation, or minus-end polymerization events during this period.

We observed MT organization in Δ*momal3*. A rigid MT network was revealed to exist during hyphal growth (Supplemental Movie S5). The mutant appears to lose most of its MT dynamics. We calculated the parameters of the MT dynamic assembly. The results showed that the MT assembly parameters were significantly lower in the transgenic plants than in the WT plants (Figure 3B–F). Additionally, the single-MT assembly and the shrinkage rates decreased markedly (Figure 3G,H).

Overall, these results revealed that *M. oryzae* employs a unique MT assembly pattern to organize a parallel MT array and a condensed hyphal tip actin during polar growth according to the observations (Figure 3I and Figure S2). MoMal3 predominantly contributed to the dynamic assembly of MTs during this process.

### 2.4. MoMal3 Is Involved in Vesicle Trafficking and Actin Organization

**Figure 4:**
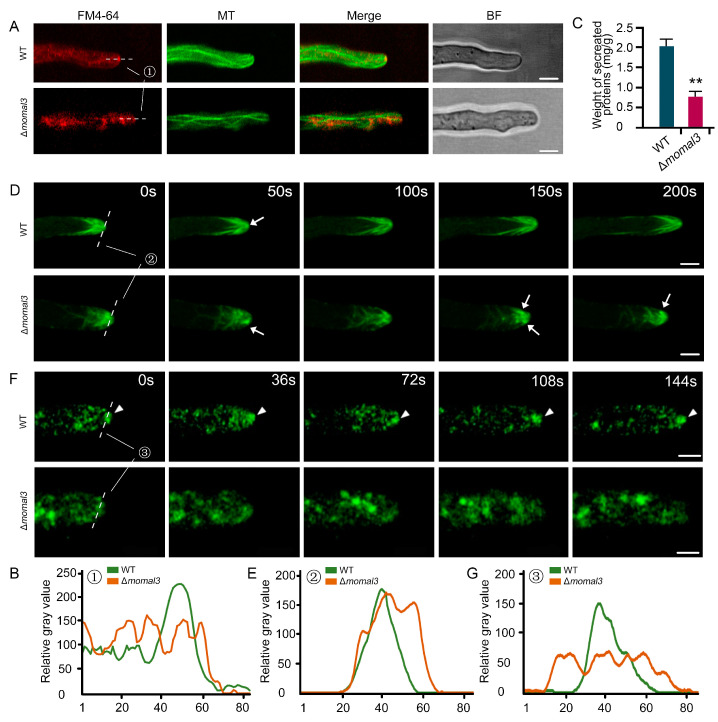
Defects in vesicle polar trafficking and actin organization in Δ*momal3.* (**A**) Representative images showing FM4-64-stained hyphae of the WT and Δ*momal3* strains. Bars = 5 μm. Thirty cells were observed for both the WT and Δ*momal3* strains, and similar observations were made. (**B**) Line-scan analysis of FM4-64 aggregation at the hyphal tip region ① shown in (**A**). (**C**) Analysis of protein secretion in the Δ*momal3* strain. The error bars show the means ± SDs of three biological repetitions of the experiment. Asterisks indicate statistically significant differences according to Student’s *t* test (**, *p* < 0.01). (**D**) Actin organization in the WT and Δ*momal3* growing hyphae. The arrows indicate actin at the Spk. The corresponding movie is provided as Supplemental Movie S6. Bars = 5 μm. Fifty WT and Δ*momal3* cells were observed, and similar observations were made. (**E**) Line-scan analysis of actin at the Spk at the hyphal tip region ② shown in (**D**). (**F**) Distribution of the GFP-labelled v-SNARE protein Snc1 in the hyphae of the WT and Δ*momal3* plants. The arrowheads indicate Snc1-GFP distributed at the hyphal tip. The corresponding movie is provided as Supplemental Movie S7. Bars = 5 μm. Fifty WT and Δ*momal3* cells were observed, and similar observations were made. (**G**) Line-scan analysis of Snc1-GFP distributed at the hyphal tip region ③ shown in (**F**).

Knockout of *MoMal3* results in defects in hyphal polar growth (Figure S3K). We next investigated whether polar trafficking of the vesicles was affected in the Δ*momal3* strain. We stained the hyphae of the WT and Δ*momal3* strains with the endocytosis marker FM4-64. Approximately 5 min after incubation with FM4-64, the red signals were endocytosed in the cytoplasm of the hyphae and delivered to the hyphal tip, where the Spitzenkörper (Spk) localized, and some MTs could directly connect to this vesicle accumulation center (Figure 4A). However, in the Δ*momal3* strain, the transport of FM4-64 to the hyphal tip was attenuated (Figure 4A). Line-scan analysis further showed that, compared with that in the WT, the transport of vesicles to the hyphal tip region in the Δ*momal3* strain was impaired (Figure 4B). We also examined the protein secretion ability of the WT and Δ*momal3* strains. We found that, compared with the WT strain, the Δ*momal3* strain exhibited an approximately 60% reduction in protein secretion (Figure 4C).

The actin cytoskeleton is crucial for *M. oryzae* polar growth and vesicle transport. We then labelled *M. oryzae* with lifeact-GFP, a widely used actin labelling marker. A dense actin network was organized at the growing hyphal tip. A bright actin aggregation spot localized at the Spk region and actin cables originating from the Spk formed long actin bundles radiating into the cell cytoplasm (Figure 4D; Supplemental Movie S6A). However, in the Δ*momal3* strain, we observed that actin organized into net-like structures. In particular, actin at Spk cannot be maintained at the extreme hyphal tip. Scattering or swaying near the hyphal tip was observed (Figure 4D; Supplemental Movie S6B). The line-scan analysis further supported the confusing organization of actin in the Spk region (Figure 4E).

Protein secretion and actin organization were both affected in the Δ*momal3* strain, indicating that the dynamic polar transport of vesicles to the hyphal tip may be disrupted. To verify this, we introduced Snc1-GFP (Snc1, a putative vesicle-bound v-SNARE protein) into the WT and Δ*momal3* strains. In the WT, Snc1-GFP-labelled vesicles were actively transferred and aggregated at the hyphal tip (Figure 4F, Supplemental Movie S7A). However, in the Δ*momal3* strain, there was no obvious accumulation of these vesicles at the hyphal tip (Figure 4F; Supplemental Movie S7B). Line-scan analysis verified the abnormal distribution of vesicles in the Δ*momal3* strain (Figure 4G).

Taken together, these results indicate that vesicle polarized transport and protein secretion were impaired in the Δ*momal3* strain.

### 2.5. MoMal3 Is Required for Penetration and Expansion in Host Plant Cells

**Figure 5:**
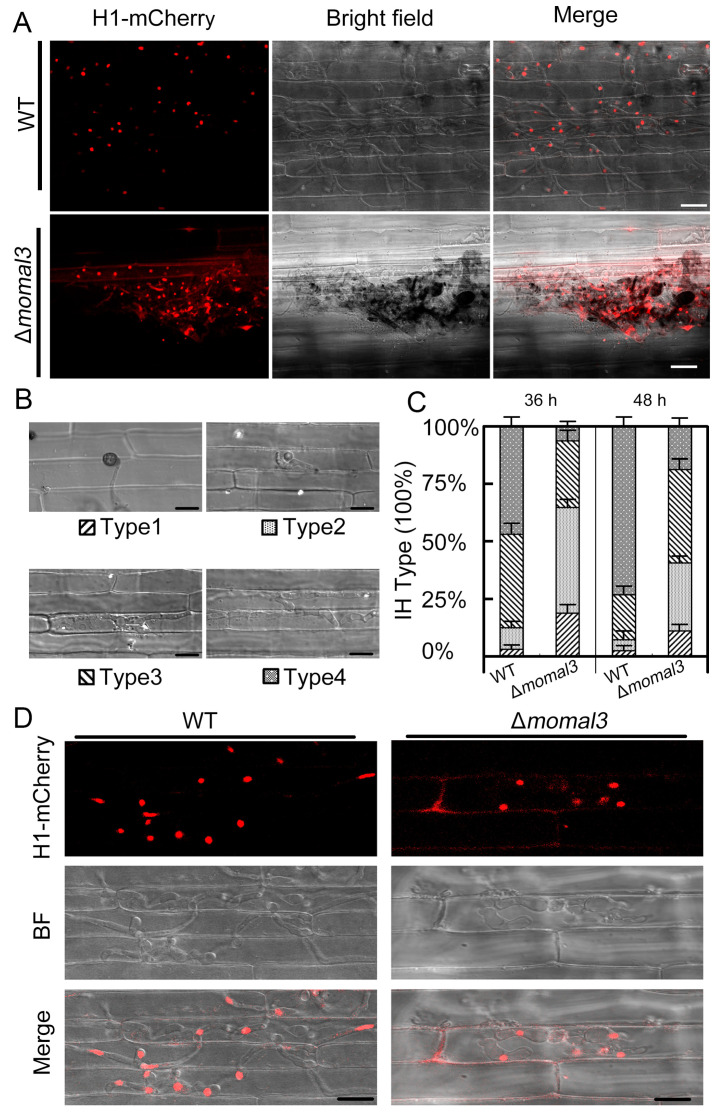
Penetration analysis of Δ*momal3.* (**A**) Rice leaf sheath cells were inoculated with H1-mCherry-labelled WT or Δ*momal3 M. oryzae* spores. Photographs were taken 72 h after infection. Scale bars = 5 µm. (**B**) Growth of invasive hyphae (IH) on rice sheath cells. Four typical types of IH were quantified and statistically analyzed for both the WT and Δ*momal3* strains at 36 h and 48 h after infection (**C**). The error bars represent the SDs; *n* ≥ 50 cells. Bar = 20 μm. (**D**) Representative images showing the infection process of WT or Δ*momal3* at 48 h after infection. WT IHs spread to neighboring rice cells, and Δ*momal3* IHs still proliferated in the primary infected cells. Bars = 20 µm.

Penetration assays using rice sheath tissues were carried out to investigate how MoMal3 functions in *M. oryzae* infection. To test this hypothesis, we introduced an mCherry-labelled histone 1 protein (H1-mCherry) into both the WT and the Δ*momal3* strains. After 72 h of infection with the conidia, the WT hyphae successfully penetrated and proliferated in the rice sheath cells (Figure 5A, upper panel). However, in the Δ*momal3* strain, most of the hyphae grew outside of the plant cells, and only approximately 30% of the Δ*momal3* appressorium could penetrate the rice cells (Figure 5A, bottom panel).

Four stages of hyphal invasion can be observed during host infection: type 1, appressorium formation; type 2, IH with fewer than two branches; type 3, IH with more than two branches; and type 4, IH penetrating neighboring cells [11]. Penetration assays were conducted by observing 200 appressoria for the WT and the Δ*momal3* strains. We classified the IH types at 36 h and 48 h after infection. We found that the progression of infection was much delayed in the Δ*momal3* strain compared with the WT strain. Even at 48 h after infection, approximately 75% of the WT IH strains progressed to stage 4, and most of the Δ*momal3* strains were in stage 2 or stage 3 (Figure 5B,C). Fluorescence observations clearly showed a typical infection event for the WT and Δ*momal3* strains. While the WT IHs penetrated multiple layers of rice cells, those in the Δ*momal3* strain still proliferated in the primary infected cells at 48 h after infection (Figure 5D).

Taken together, these results indicated that knockout of *MoMal3* strongly impaired the ability of *M. oryzae* to cause infection.

### 2.6. MoMal3 Is Critical for the Dynamic Assembly of MTs in the Appressorium

**Figure 6:**
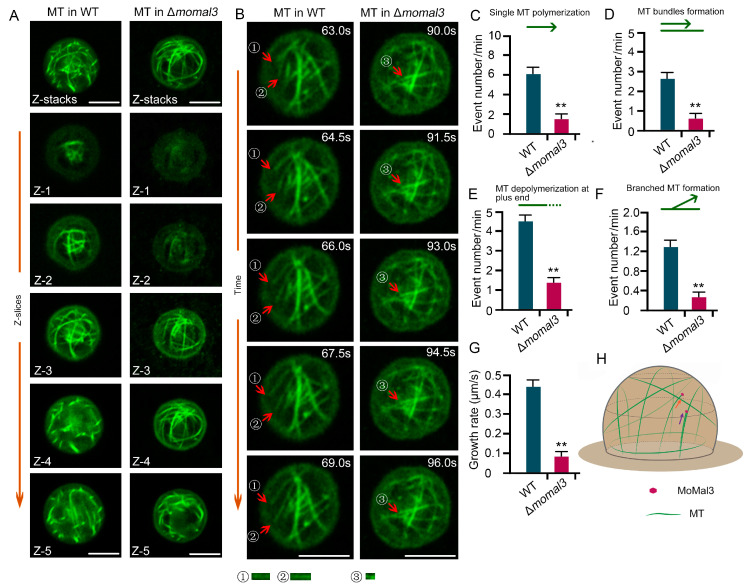
Microtubule organization in the WT and Δ*momal3* appressoria. (**A**) Maximum projection of Z-slices obtained by imaging an 8 h developed appressorium of the WT and Δ*momal3* strains illustrating the structure of the MT cytoskeleton. Thirty cells were observed for both the WT and Δ*momal3* strains. A 3D movie generated from these slices showing the spatial structure of the MT cytoskeleton in the appressorium. The corresponding movie is provided as Supplemental Movie S8. Bars = 10 μm. (**B**) Time-lapse images showing the MT dynamics in an 8 h developed appressorium of the WT and Δ*momal3* strains. Fifty WT and Δ*momal3* cells were observed, and similar observations were made. The corresponding movies are provided as Supplemental Movies S9 and S10. The red arrows indicate MT elongation events. ①–③ indicates the elongated MT at the same time period.The numbers at the top right corner indicate the timestamps (S). Bars = 10 μm. (**C**–**G**) Statistical analysis of the number of single MT polymerization events (**C**), MT bundle formation (**D**), MT depolymerization at the +tip end (**E**), branched MT formation (**F**) and the single MT elongation rate (**G**) in the appressorium of the WT and Δ*momal3* strains in Supplemental Movies S9 and S10. The green arrows indicate the MT assembly direction and pattern. And the dash line in (E) indicates the depolymerization of MT at the MT (+) plus end. The error bars represent SDs (*n* = 100), and the asterisks (**) represent significant differences (*p* < 0.01). (**H**) A proposed model showing the organization of the MT at the appressorium. Arrows indicate the MT assembly direction.

A rice infection assay showed that a majority of the Δ*momal3* appressorium failed to penetrate the plant cells. This prompted us to investigate the MT in the appressorium. Unlike parallel MT arrays in hyphae, in dome-shaped appressoria, the cortical MT aligns as net-like arrays, forming cage-like structures that confine the cell (Supplemental Movie S8). These proteins were usually assembled as curved MTs from the base to the top of the appressorium for both *M. oryzae* strains (Figure 6A). We next investigated the dynamic assembly of these vesicles using time-lapse live imaging. As the MT in the dome-shaped appressorium is highly dynamic, we cannot perform both Z-stack and time-lapse imaging at the same time to record the MT. We analyzed the dynamics of both the WT and the Δ*momal3* cells in the middle slice of the appressorium. The results showed that, as in the growing hypha, MTs in the appressorium were dynamically assembled, including through polymerization or depolymerization at the +tip end and the formation of branching MTs (Figure 6B; Supplemental Movie S9). Interestingly, although the MTs in the appressorium organized as net-like arrays, the majority of MT growth events involved single-filament polymerization and bundled MT generation (Figure 6C,D). MT +tip depolymerization also occurred at a high frequency (Figure 6E). We calculated these MT assembly events in Δ*momal3* and found that all these parameters were significantly lower than those in the WT (Figure 6C–F; Supplemental Movie S10). We also calculated the average growth rate of MTs in the Δ*momal3* strain, which was much lower than that in the WT strain (Figure 6G). A schematic diagram was drawn to show the MT organization in the *M. oryzae* appressorium according to the observations (Figure 6H). Taken together, these findings indicated that the Δ*momal3* appressorium lost much of its MT dynamics.

To investigate how a frozen MT array influences the penetration ability of the appressorium, we observed the actin organization of both the WT and the Δ*momal3* appressorium. A Lifeact-GFP-labelled ring-like structure of the actin cytoskeleton was observed in the WT appressorium (Figure S4A), but in the Δ*momal3* strain, a deformed actin structure was present in the cell (Figure S4B). Line-scan analysis further confirmed the discrepancy between the two strains (Figure S4C).

We also introduced the turgor-sensing histidine–aspartate kinase MoSln1 fused with mCherry into two *M. oryzae* strains. MoSln1 could enable the appressorium to sense the turgor threshold and intrigue the appressorium to penetrate host cells. The turgor threshold can be sensed, and the fungus can gradually accumulate in the appressorium pore with increasing turgor pressure during appressorium maturation [9]. We found that when MoSln1-mCherry was distributed at the appressorium pore in the WT strain at 24 h after conidia had germinated on glass coverslips, MoSln1-mCherry was still localized at the periphery of the cell membrane (Figure S4D,F), indicating decreased turgor pressure in the cell.

### 2.7. Knockout of MoMal3 Results in Defects in Nuclear Division

**Figure 7:**
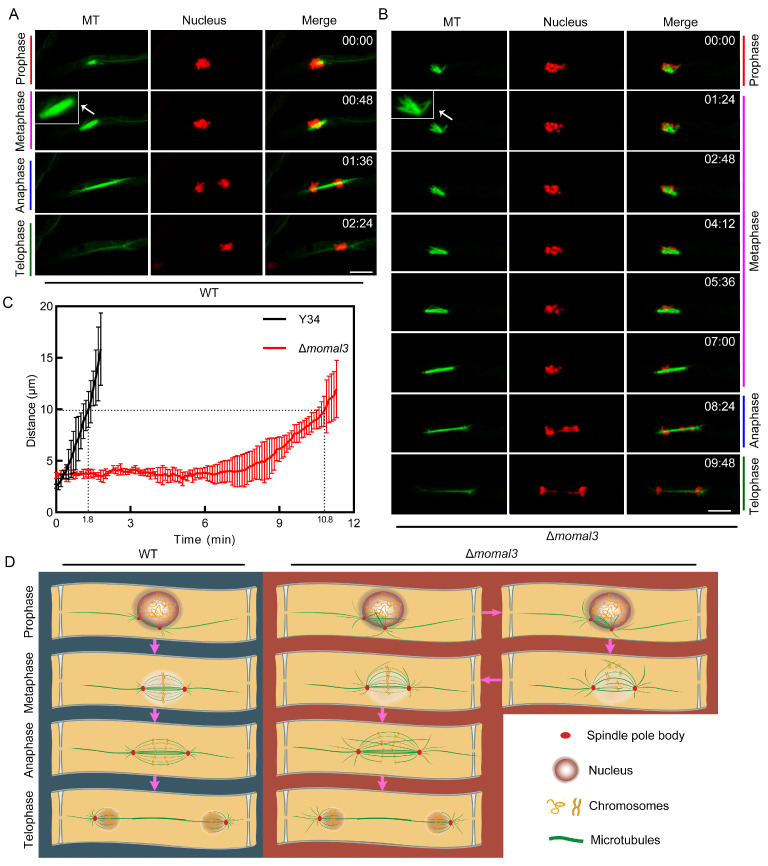
MT spindle and nucleus division in the hyphae of the WT and Δ*momal3* strains. (**A**,**B**) Time-lapse images showing the MT dynamics and nuclear division in the hyphae of the WT (**A**) and Δ*momal3* (**B**) strains. Thirty cells were observed for both the WT and Δ*momal3* strains, and similar observations were made. Histone1-mCherry was expressed in the MT-labelled WT and Δ*momal3* strains. The four mitosis stages are shown in the figure for both the WT and the Δ*momal3* strains. The numbers at the top right corner indicate the timestamps (Min:Sec). The corresponding movie is Supplemental Movie S11. Bars = 5 μm. The white arrows indicate the enlarged spindle. (**C**) Normalized curve showing the chromosomal distance in mitosis shown in (**A**,**B**). (**D**) A diagram showing the development of the MT spindle related to the division of the chromosomes in the WT and Δ*momal3* strains. Pink arrows indicate the mitotic progressions.

MoMal3 also localized to the MT spindle during fungal mitosis (Figure 2B), indicating that it may function in nuclear division. To test this possibility, we investigated nuclear division using both the MT and nucleus-labelling (H1-mCherry) strains for the WT and Δ*momal3* vegetative hyphae. Time-lapse imaging experiments showed that *M. oryzae* could rapidly complete nuclear division. Approximately 2–3 mins were required for the WT to complete the whole process from prophase to telophase. We observed that the MT gradually assembled a stick-shaped spindle during prophase and metaphase and elongated rapidly during anaphase and telophase. Along with the changes in the MT spindle, the chromosomes were condensed, aligned and separated during this process (Figure 7A, Supplemental Movie S11A). We also monitored division in the Δ*momal3* strain, and the results revealed that a much longer time (approximately 10 min) was required for the Δ*momal3* strain than for the WT strain to complete the whole nuclear division process (Figure 7B). The increase in the duration of nuclear division was attributed mainly to the delay of prophase and metaphase in the Δ*momal3* strain. These two periods were sustained for approximately 7 min for Δ*momal3*, but for the WT, they were less than one minute (Figure 7A,B; Supplemental Movie S11B). The nucleus division curve corresponding to Supplemental Movie S11 directly presented discrepancies between the two strains. Compared with the WT, Δ*momal3* needed more time to separate the duplicated chromosomes to a certain distance (10 µm) (Figure 7C).

Compared with those of the WT, the Δ*momal3* strain formed a loose MT spindle at prophase and metaphase. Some of the straggly MTs were assembled and extended from the spindle orming an unsymmetrical spindle body (Figure 7B). We constructed a schematic diagram to show the course of nuclear division according to the observations of the WT and Δ*momal3* strains. The results showed that Δ*momal3* assembled a deformed MT spindle, and more time was needed to align and separate the chromosomes than was needed for the WT (Figure 7D).

### 2.8. MoMal3 Is Important for M. oryzae Proliferation in Host Plant Cells

**Figure 8:**
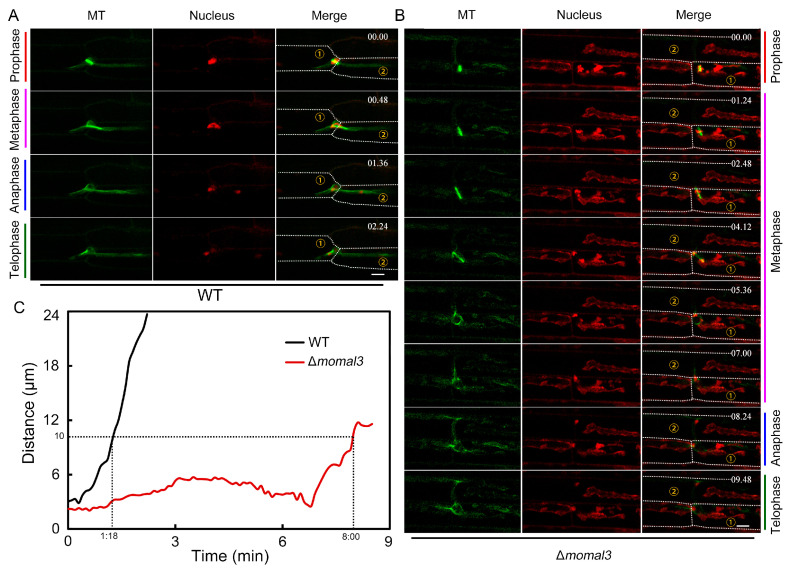
Nucleus division and transfer to neighboring cells during WT and Δ*momal3* infection. (**A**,**B**) Timelapse images showing the MT dynamics and nuclear division in the invasive hyphae of the WT (**A**) and Δ*momal3* (**B**) strains. Twenty-five cells were observed for both the WT and Δ*momal3* strains, and similar observations were made. Histone1-mCherry was expressed in the MT-labelled WT and Δ*momal3* strains. The four mitosis stages are shown in the figure for both the WT and the Δ*momal3* strains. In the prophase of the WT plants, the nucleus was arranged close to the plant cell wall. In addition, during metaphase, the *M. oryzae* MT spindle crosses the plant cell wall and begins to draw chromosomes into neighboring plant cells. However, in the Δ*momal3* strain, the time required for the MT spindle to cross the plant cell wall was longer. The numbers at the top right corner indicate the timestamps (Min:Sec). The corresponding movie is provided as Supplemental Movie S12. Bars = 2 μm. ① means the primary infected cell and ② means the neighbouring infected cell. (**C**) Normalized curve showing the chromosome distance in mitosis shown in (**A**,**B**).

The above-described findings indicated that nuclear division is abnormal in the Δ*momal3* vegetative hyphae, and we next investigated the nuclear behavior of the invasive hyphae. Fluorescently labelled *M. oryzae* for both the MT and nucleus were used to infect the rice sheath cells. Time-lapse imaging revealed four typical stages of nuclear division in the invasive hyphae. These separated nuclei either remained in the primary rice cell or were transported to the neighbor. The images showed that the divided chromatin of the WT was transported into the neighboring cell (Figure 8A). In the prophase of the WT cells, the MTs formed short stick-shaped spindles, and the duplicated chromosomes were condensed and close to the rice cell wall. At metaphase, the MT spindle elongates and extends across the plant cell wall, and the chromosomes are aligned and begin to migrate to adjacent plant cells. Subsequently, at anaphase and telophase, the chromosomes were rapidly separated into adjacent rice cells (Supplemental Movie S12A).

We then observed the mitotic migration of Δ*momal3* nuclei during plant infection. Like in vegetative hyphae, the whole nuclear division and migration cycle took a much longer time in the Δ*momal3* strain than in the WT strain (Figure S5). Prophase and metaphase were significantly longer (approximately 7 min) in these plants than in the WT plants (less than 1 min) (Figure 8B,C). The stick-shaped spindle in Δ*momal3* appears to take a longer time to extend across the rice cell wall than that in the other strains, after which the penetration of chromosomes near the plant cell wall is delayed (Supplemental Movie S12B). Taken together, these results indicated that knockout of *MoMal3* leads to defects in *M. oryzae* proliferation in host plant cells.

## 3. Discussion

Research associated with blast fungus infection is one of the most popular topics in plant disease investigations [30]. Currently, *M. oryzae* is considered a model organism for revealing important concepts related to filamentous fungus development and fungal–plant interactions [1]. Although the organization of MT networks plays critical roles in controlling different aspects of cell architecture and function, we did not fully understand the assembly aspects associated with MT arrays or the dynamic assembly parameters or those linked to their cellular functions in the development and infection of the model filamentous fungus *M. oryzae*. In this study using high-resolution live-cell imaging, we investigated the organization and assembly pattern of MT during *M. oryzae* hyphal polar growth, appressorium formation and cell proliferation during plant host cell penetration. We revealed that MT dynamically assembles into different arrays during the *M. oryzae* growth and infection cycle and showed that these arrays are pivotal for *M. oryzae* development and host infection.

Growth of *M. oryzae* hyphae occurs at the cell apex through the polarized secretion of materials required for sustaining tip expansion. This process is referred to as tip growth and is a pattern analogous to plant pollen tube growth and root hair elongation [31]. In plant pollen tube cells, long and axially oriented thick MT bundles were found in the distal region of the pollen tube, whereas randomly oriented short MT fragments existed in the apical or subapical regions [32,33]. Dense cortical MTs also appeared in growing root hairs. They are oriented net-axially in the shank, but whether they reach the very tip [34] has been debated for a long time. Like in these cells, an axially oriented MT array was also organized in *M. oryzae*-growing hyphae (Figure 3A; Supplemental Movie S4), but these MTs were mostly assembled as linear filaments, and crossed MTs were rarely observed. We speculated that such a linear MT array may greatly facilitate hyphal tip growth. In the hyphae of the fungus *N. crassa*, MTs exhibit a helical curvature with a long pitch and a tendency to intertwine with one another to form a loosely braided network throughout the cytoplasm, and a ring-like arrangement of MT segments surrounding Spk is usually observed at the hyphal tip [19]. However, in *M. oryzae*, we did not observe such an MT structure at the hyphal tip. Instead, some MT filaments could directly connect to the Spk at the hyphal tip (Figure 4A). Fungal MTs are considered to serve as tracks for secretory vesicles for long-distance transport to hyphal tips [35]. These Spk-connected MTs may directly deliver vesicles to Spk and facilitate protein secretion.

The MT cytoskeleton plays a decisive role in controlling cell expansion and driving cell morphogenesis [36]. Especially in plant cells, the orientation of the cortical MT array controls cell anisotropy by guiding the deposition of cellulose synthase complexes along MTs [37]. Unlike hyphae, which have a unique growth direction, *M. oryzae* can develop a unicellular dome-shaped appressorium that is isotropic partly to generate enormous turgor, which is translated into mechanical force to penetrate plant cells. It has been revealed that some MT loops appear at the base of an immature appressorium. These cells were assembled as vertically orientated polarized microtubule arrays, with their plus ends toward the appressorium pore [15]. In this study using high-resolution imaging, we further found that in the appressorium, curvilinear MTs usually align to form net-like arrays (Figure 6; Supplemental Movie S8). This configuration was similar to the MT configuration in an initiated cotton fiber cell, which is also dome-shaped. In an initiated cotton fiber cell, the MTs were assembled haphazardly, and such MT organization was required for the generation of intracellular turgor pressure, facilitating cotton fiber elongation [38]. Thus, we speculated that the organization of a stereoscopic net-like MT array may also play a role in maintaining the spheroidal morphology and generating turgor in the *M. oryzae* appressorium.

Eukaryotic cells remodel their MT network in a regulated manner to form various arrays. Nucleation, elongation, bundling, capping, serving and catastrophe are all critical steps for MT remodeling [39]. These events for the dynamic assembly of MTs in plant pathogenic fungi are not clear in the life cycle of these fungi. We found that in *M. oryzae* hyphae and appressoria, +tip MT polymerization and depolymerization accounted for the majority of the MT assembly events. MT branching and serving are rare (Figure 3 and Figure 6), indicating that *M. oryzae* employs a unique MT assembly pattern from other life cells.

Like the fungus *A. nidulans* [21], the Δ*momal3* hypha exhibited an undulating pattern instead of unidirectional growth in the control, and the growth rate was greatly reduced (Figure S3). Imaging of the MT dynamics showed that the MT growth rate, MT polymerization and depolymerization frequency in the Δ*momal3* strain were significantly decreased, indicating the organization of a frozen MT network (Figure 3B–G). However, it is unclear how aberrant MT organization results in altered directional and polar growth. EB1 proteins can link microtubules and the actin cytoskeleton via specific cellular domains [40,41]. In the hyphal tip of Δ*momal3*, the actin organization was changed. Mesh-like rather than straight actin cables connecting to Spk could be observed in the hyphal subapex of the Δ*momal3* and the control strains. In particular, the actin at Spk in Δ*momal3* could not be maintained at the hyphal tip (Figure 4D). Thus, we speculate that MoMal3 could also function in linking microtubules and the actin cytoskeleton at the hyphal tip. Additionally, knockout of *MoMal3* results in disordered actin organization at the hyphal tip, which attenuates hyphal placental growth and protein secretion. The swaying motion of Spk leads to the waving growth of the Δ*momal3* hyphae.

However, it is unclear how MTs are organized and their functions in the infection structure of *M. oryzae*. Disruption of MT using benomyl has been shown to result in fragmentation of the septin disc at the appressorium pore [15], indicating functional cooperation between the MT, actin and septin cytoskeletons. In the Δ*momal3* strain, MT dynamics were markedly impaired (Figure 6B; Supplemental Movie S10), and the ability to penetrate the appressorium was severely reduced (Figure 5A). Cytorrhysis and distribution of the turgor sensor protein MoSln1 showed that the turgor pressure was not comparable to that of the WT (Figures S3N and S4D,E). In addition, in the Δ*momal3* appressorium, a ring-like actin structure could not form at the base of the cell where the penetration peg initiates (Figure S4B,E). Based on these findings, we speculate that, on the one hand, the cage-like MT array confines the cell, helping to generate turgor in the cell. Future studies on the MT orientation transition during appressorium maturation will further reveal the association between the MT array and turgor generation. On the other hand, MTs may also help with the formation of ring-like actin structures at the cell base through interactions with the actin and septin proteins.

MoMal3 also exhibited spindle MT localization, indicating the function of MoMal3 in nuclear division (Figure 2B; Supplemental Movie S3). In the vegetative hyphae, compared with those of the WT, the Δ*momal3* strain assembled a loose MT spindle at prophase and metaphase (Figure 7), the time at which the chromosomes were aligned [42]. An abnormal spindle in the Δ*momal3* strain hindered nuclear division (Figure 7D). We speculate that this effect may be involved in the inappropriate attachment of sister kinetochores to the spindle. Nuclear division plays crucial roles in penetration and cell-to-cell spread in host cells [5]. It would be interesting to consider how the nucleus (~2 μm diameter) migrates through the constricted invasive hyphal peg (~0.5 μm diameter) [43]. It has been indicated that in invasive hyphae, the divided nucleus can be squeezed to a diameter less than 0.5 μm to pass through the narrow invasive hyphal peg and pit fields of the plant cell wall [44]. In this process, the nucleus undergoes extreme compression and then migrates to adjacent host plant cells [45]. In our study, high-resolution imaging provided additional details about this process. We found that nuclear migration is accompanied by mitosis. In a typical wild-type cell, the duplicated chromosomes were arranged close to the plant cell wall at prophase and metaphase. At this time, the stick-shaped MT spindle crossed the narrow invasive hyphal peg and pit fields on the cell wall between the two plant cells. Along with the elongation of the spindle, the chromosomes were pulled and rapidly separated into neighboring plant cells (Figure 8A; Supplemental Movie S12). Furthermore, we found that MoMal3 was required for this process. During the prophase and metaphase of the Δ*momal3* strain, the assembled spindle required a longer time to cross the invasive hyphal peg and pit field on the plant cell wall. The loose spindle appears to need more time to adjust its morphology and direction to pass through the channel (Figure 8B; Supplemental Movie S12).

Rice blast disease is one of the most significant threats to global food production. Insight into the underlying cellular mechanisms is important for controlling devastating rice blast fungus both theoretically and in practice. Our study provides new evidence that, like the actin cytoskeleton, the MT is also specifically organized and plays crucial roles in *M. oryzae* development and pathogenesis. Considering that multiple proteins can regulate many aspects of MT organization and function, we believe that future work on *M. oryzae* MT will provide additional information to clarify its infection mechanism and provide new targets for fungicide design.

## 4. Materials and Methods

### 4.1. M. oryzae Strains and Culture Conditions

*M. oryzae* Y34 was used as the wild type in this study, and all the *M. oryzae* Y34 knockouts and transformants had a Y34 background. After being cultured in liquid complete medium (CM) at 28 °C for two days, the hyphae were harvested, and DNA was extracted [46]. The strains used for the growth and infection experiments were cultured on agar CM and straw rice bran (SRB) media at 28 °C for 5–15 days. Protoplast production and transformation were performed as described previously [47].

### 4.2. Virulence Assay

Virulence assays were performed according to previous methods [48]. The spores cultured on SRB media were collected and diluted with 0.02% Tween solution to a concentration of 5 × 10^4^ spores/mL. For spray infection, two-week-old *Oryza sativa* cv. Nipponbare plants were selected, and 10 mL of the spores were evenly sprayed on the leaves with a sprayer. For puncture infection, rice seedlings were selected for approximately 4–5 weeks, the leaves were punched into a round wound, 10 μL of spore solution was added to the wound, and the wound was sealed with transparent tape. The inoculated rice plants were cultured at 26 °C and 90% humidity in the dark for 24 h and then put into a rice growth incubator. For leaf sheath infection, the rice leaf sheath was cut into 4–8 cm pieces. The *M. oryzae* spore solution was injected into the leaf sheath with a syringe needle and placed in a closed and dark environment at 28 °C and 90% humidity. The inoculation results were observed by optical microscopy.

### 4.3. Developmental Analysis of Hyphae, Conidia and Appressoria

For the colony growth assay, the same amount of conidia of Y34, Δ*momal3* and the complemented strain were cultured in the center of the media and cultured in the dark at 28 °C. The colony radius was measured with a Vernier calliper every day. For the examination of appressorium formation, the spore suspension was placed on a hydrophobic slide. After 12 h of aseptic culture, the appressorium formation efficiency of Y34, Δ*momal3* and the complemented strain was compared and calculated [11].

### 4.4. FM4-64 Staining and Secreted Proteins Extracted from M. oryzae Hyphae

The *M. oryzae* hyphae were stained with FM4-64 (Thermo Fisher T3166, Waltham, MA, USA) at a concentration of 10 μM in the dark. Then, the fluorescence was observed under a confocal microscope (Zeiss, LSM880, Oberkochen, Germany). To extract the secreted proteins from *M. oryzae*, fresh WT and *MoMal3* mutant mycelia were cultured in liquid CM for 48 h. Then, the mycelia were harvested by filtration, and an equal weight of WT and *MoMal3* mutant mycelia was transferred to liquid GMM for 24 h [49]. The secreted proteins in the medium were collected and condensed in an ultrafiltration tube (3 kD, Millipore, UFC8030, Billerica, MA, USA). Then, the protein concentrations were quantified by the Bradford method.

### 4.5. Targeted MoMal3 Deletion and Plasmid Construction

The deletion of *MoMal3* in *M. oryzae* was performed using a previously reported method [50]. To obtain the complemented strain, DNA fragments containing the 1.5-kb native promoter region of *MoMal3* were ligated and cloned and inserted into the pKNTG binary vector. To construct plasmids expressing MoMal3-mCherry and SNC1-GFP, the related DNA fragments from the *M. oryzae* genome and the ~1.5-kb native promoter region were amplified and cloned and inserted into the pKNTG binary vector. To construct the Histone1-mCherry, NLS-mCherry, Lifeact-GFP and β-tubulin plasmids, the corresponding DNA sequences were ligated to GFP and cloned and inserted into the PsulPH vector [7,51,52]. All primers with restriction enzyme sites are listed in Table S1 in the Supporting Information. The recombinant plasmids were subsequently transformed into *M. oryzae* protoplasts, as described previously [47].

### 4.6. Observation of Fluorescent Signals by High-Solution-Resolution Live-Cell Imaging

Live-cell imaging was performed under a high-resolution confocal microscope (Zeiss LSM880) equipped with an Airyscan detector, and fluorescence images were acquired with a 60 × 1.4 oil objective. The images were processed and analyzed using ImageJ (v1.8.0) (http://rsbweb.nih.gov/ij (accessed on 12 December 2023)), as previously described [15]. The maximum projection of the image stack was used to record the global organization of the MT cytoskeleton.

### 4.7. Accession Numbers

Sequence data for the genes described in this study can be found in the GenBank/EMBL database under the accession numbers MoMal3 (ELQ35583), Snc1 (ELQ36245), and Sln1 (ELQ34371.1).

## Data Availability

The data presented in this study are available in “The microtubule end binding protein Mal3 is essential for dynamic microtubule assembly during *Magnaporthe oryzae* growth and pathogenesis”.

## Supplementary Materials

The following supporting information can be downloaded at: https://www.mdpi.com/article/10.3390/ijms25052672/s1.
