# Supplementary material for: The Microtubule End Binding Protein Mal3 Is Essential for the Dynamic Assembly of Microtubules during Magnaporthe oryzae Growth and Pathogenesis
Source: Int J Mol Sci. 2024 Feb 26;25(5):2672. doi: 10.3390/ijms25052672 (PMC10932144; doi:10.3390/ijms25052672)
Supplement: Supplementary file 1 [file ijms-25-02672-s001.zip › ijms-2873789-supplementary.pdf]

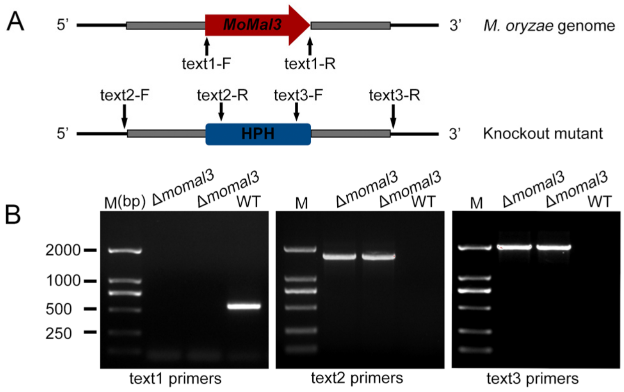

Figure S1. Construction of the MoMal3 knockout mutant

(**A**), Schematic representation of the recombination event involved in the targeted replacement of *MoMal3*. (**B**), PCR identification of the knockout mutant using the primers indicated in (**A**). Lanes 1, 2, and 3 indicate the *MoMal3-1*, *MoMal3-2* mutant and the WT strain, respectively.

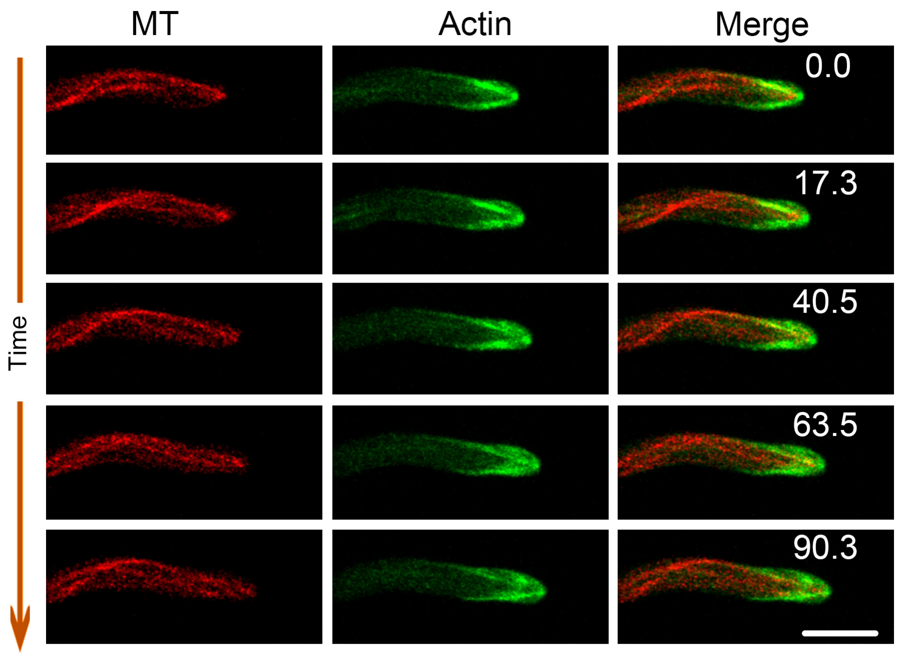

Figure S2. Actin and microtubule cytoskeletons of growing hyphae

Time-lapse imaging of the MT and actin assembly in the WT *M. oryzae* growing hypha. This representative video is based on data from 20 hyphae in three independent experiments. The numbers at the top right corner indicate the timestamps (S). Bar = 5 μm.

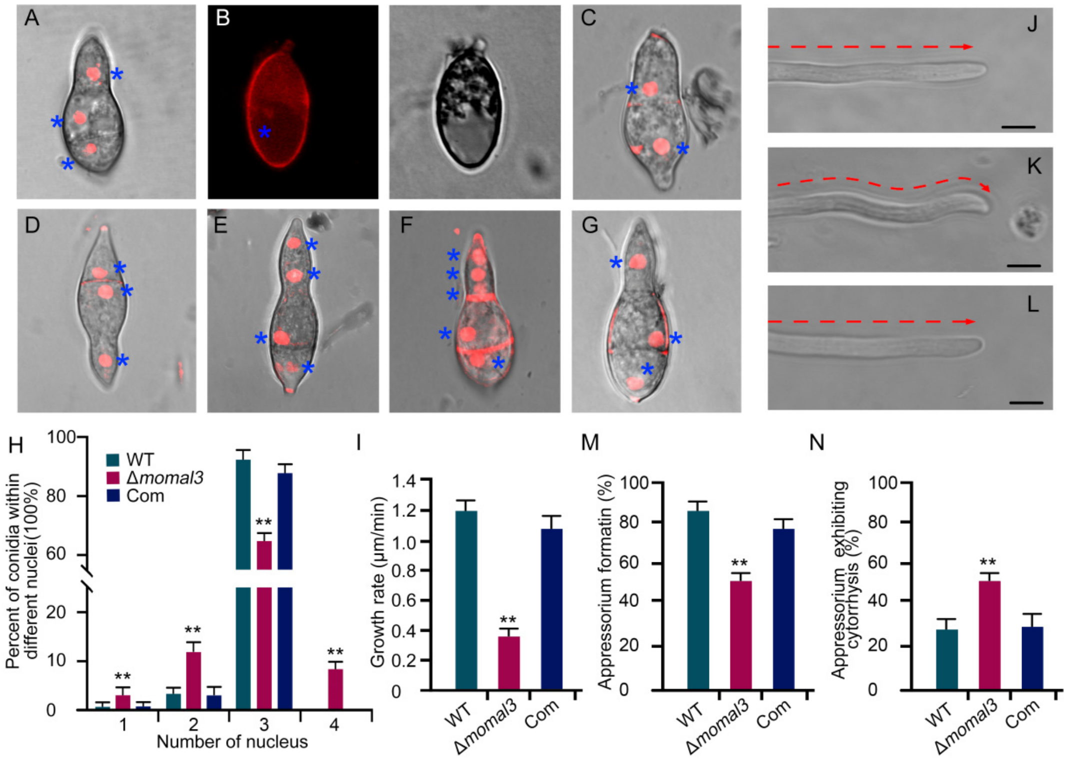

Figure S3. Development defects of Δ*momal3*

(**A**–**G**), Photographs of the NLS-mCherry labeled nuclei in the conidia of WT (**A**), Δ*momal3* (**B**–**F**) and the complemented (**G**) strains. The nucleus labeling marker NLS-mCherry was expressed in the WT, Δ*momal3* and the complemented strains. (**H**), Statistics analysis of the number of the nuclei in the WT, Δ*momal3* and the complemented strains. Error bars represent SD (n = 200) and asterisks (**) represent a significant difference ($p < 0.01$). Bars = 5 μm. (**I**), Hyphal growth speed of the WT, Δ*momal3* and the complemented strains. 100 hyphae each of the WT, Δ*momal3*, and the complemented strain were calculated in three experimental repeats. The corresponding movie is provided as Supplemental movie S1. Error bars represent SD (n = 100). Asterisks indicate statistically significant differences, as determined by Student's t-test ($p < 0.01$). (**J**–**L**), Representative images showing the morphology of the WT (**J**), Δ*momal3* (**K**) and the complemented strains (**L**). Bars = 5 μm. (**M**), Appressorium formation rates were calculated and statistically analyzed. Error bars represent SD (n = 100). Asterisks represent significant differences ($p < 0.01$). (**N**), Appressorium turgor was measured by an incipient cytorrhysis (cell collapse) assay

using glycerol (3 M). The percentage of collapsed appressoria was recorded by observing at least 100 appressorium for each strain, and were repeated for three times. Error bars represent SD and asterisks represent significant differences ($p < 0.01$).

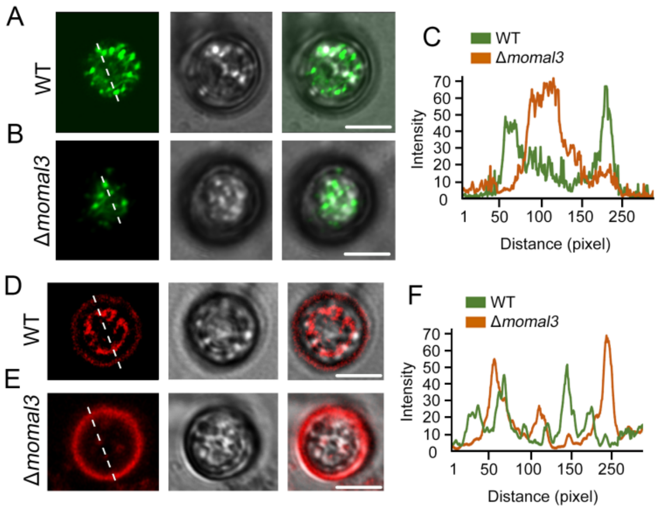

Figure S4. Actin and Sln distribution in WT and Δ*momal3* appressorium

(**A,B**), Representative image showing the actin organization in a 8 h developed WT (**A**) and Δ*momal3* (**B**) appressorium. 30 cells were observed for both WT and Δ*momal3* and similar observations were collected. Bars = 10 μm. (**C**), Line-scan analysis of the actin organization in the appressorium in (**A** and **B**). (**D**,**E**), Representative image showing the distribution of the turgor sensor protein MoSln in the 24 h developed appressorium of WT and Δ*momal3*. 30 cells were observed for both WT and Δ*momal3* and similar observations were collected. Bars = 10 μm. (**F**). Line-scan analysis of the MoSln1-mCherry in the appressorium shown in (**D** and **E**).

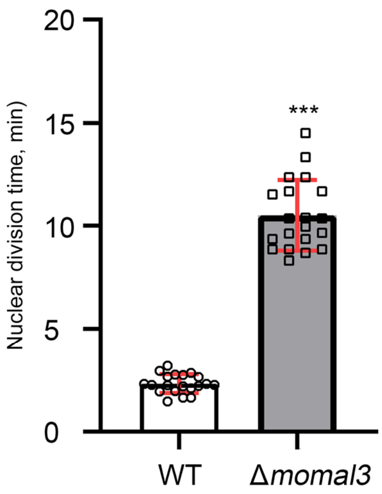

Figure S5. Statistical analysis of the nuclear division and migration time of the WT and Δ*momal3* strains.

20 nuclear division and migration events were observed for both WT and Δ*momal3* . in three experimental repeats. Error bars represent SD. Asterisks indicate statistically significant differences, as determined by Student's *t*-test ($p < 0.001$).

Supplemental movie legends

Supplemental movie S1. Hyphal growth analysis of the Δ*momal3* by microscopy

The hyphae of the WT (**A**), Δ*momal3* (**B**) and the complemented (**C**) strains were observed under a microscope (Zeiss LSM880, with a 20x objective). Time-lapse imaging was conducted and the hyphal growth rate was calculated according to the distance it grew. This representative video is based on data from 20 hyphae in three independent experiments. The numbers at the top right corner indicate the timestamps (min:sec). Bar = 20 μm.

Supplemental movie S2. MoMal3 localizes to the MT +tip end in during hyphal tip growth

Tubulin-GFP (**A**) and MoMal3-mCherry (**B**) driven by its native promoter were coexpressed in Δ*momal3*. The white and yellow arrows indicate that MoMal3 guides +tip MT polymerization along a existing MT to form MT bundles and typical single MT +tip elongation, respectively. The purple arrow and asterisk indicate that MoMal3-mCherry dose not localize to a depolymerized MT +tip and MT -tip end, respectively. This representative video is based on data from 50 hyphae in three independent experiments. The numbers at the top right corner indicate the timestamps (S). Bar = 5 μm.

Supplemental movie S3. MoMal3 localizes to the MT spindle during mitosis

Time-lapse imaging the fluorescence of the MoMal3-mCherry during *M. oryzae* mitosis. Tubulin-GFP (**A**) and MoMal3-mCherry (**B**) were coexpressed in Δ*momal3*. This representative video is based on data from 30 hyphae in three independent experiments. The numbers at the top right corner indicate the timestamps (S). Bar = 5 μm.

Supplemental movie S4. The dynamic organization of the MT cytoskeleton during hypha polar growth in the WT *M. oryzae*

Time-lapse imaging of the MT assembly in the WT growing hypha. The red and white

arrows indicate the polymerization or depolymerization of the MT at the +tip end, respectively. This representative video is based on data from 50 hyphae in three independent experiments. The numbers at the top right corner indicate the timestamps (S). Bar = 5 μm.

Supplemental movie S5. The dynamic organization of the MT cytoskeleton during hypha polar growth in Δ*momal3*

Time-lapse imaging of the MT assembly in the Δ*momal3* growing hypha. The red and white arrows indicate the polymerization or depolymerization of the MT at the +tip end, respectively. The yellow arrowheads indicate the MT catastrophe events. This representative video is based on data from 50 hyphae in three independent experiments. The numbers at the top right corner indicate the timestamps (S). Bar = 5 μm.

Supplemental movie S6. Actin organization in the hyphal tip of Δ*momal3*

Lifeact-GFP was expressed in the WT and the Δ*momal3*. Time-lapse observations were preformed to record the actin dynamic organization in the hyphal tip of WT (**A**) and Δ*momal3* (**B**). This representative video is based on data from 30 hyphae in three independent experiments. The numbers at the top right corner indicate the timestamps (Min:Sec). Bar = 5 μm.

Supplemental movie S7. Distribution analysis of the v-SNARE protein Snc1-GFP in Δ*momal3*

Snc1-GFP driven by Snc1 promoter was expressed in both WT and Δ*momal3*. Time-lapse images were obtained to record the distribution of Snc1-GFP in the hyphal tip of WT (**A**) and Δ*momal3* (**B**). The white arrowhead indicates Snc1-GFP could be transferred and aggregated at the WT hyphal tip during polar growth. This representative video is based on data from 30 hyphae in three independent experiments. The numbers at the top right corner indicate the timestamps (Min:Sec). Bar = 5 μm.

Supplemental movie S8. 3D reconstruction of MT organization in *M. oryzae* appressorium

This representative video is based on data from Z-slices of imaging of the MT in the WT (**A**) and Δ*momal3* (**B**) in Figure 7A.

Supplemental movie S9. The dynamic organization of the MT cytoskeleton in the WT appressorium

Time-lapse imaging of the MT assembly in the WT appressorium. The red and white arrows indicate the polymerization or depolymerization of the MT at the +tip end, respectively. This representative video is based on data from 50 hyphae in three independent experiments. The numbers at the top right corner indicate the timestamps (Sec). Bar = 5 μm.

Supplemental movie S10. The dynamic organization of the MT cytoskeleton in the Δ*momal3* appressorium

Time-lapse imaging of the MT assembly in Δ*momal3* appressorium. The red and white arrows indicate the polymerization or depolymerization of the MT at the +tip end, respectively. This representative video is based on data from 50 hyphae in three independent experiments. The numbers at the top right corner indicate the timestamps (Sec). Bar = 5 μm.

Supplemental movie S11. MT spindle and nucleus division in *M. oryzae* mitosis

Time-lapse imaging of both the Tubulin-GFP and Histone1-mCherry in WT (**A**) and Δ*momal3* (**B**) to show the developing of the MT spindle and the separation of the chromosomes in mitosis. The white arrow indicates separation of the spindle pole body. This representative video is based on data from 50 hyphae in three independent experiments. The numbers at the top right corner indicate the timestamps (Min:Sec). Bar = 5 μm.

Supplemental movie S12. MT spindle and nucleus division in *M. oryzae* infection of the host plants

Time-lapse imaging of both the Tubulin-GFP and Histone1-mCherry in WT (**A**) and Δ*momal3* (**B**) to show the developing of the MT spindle and the separation of the chromosomes in mitosis during *M. oryzae* spread in the host plant cells. In the infection initiation, the nucleus was arranged close to the plant cell wall and subsequently, the MT spindle crossed the plant cell wall. Accompanied with the elongation of the MT spindle, the chromosomes were pulled into the neighbour plant cell. The white arrow indicates separation of the spindle pole body. This representative video is based on data from 50 hyphae for both the WT and Δ*momal3* in three independent experiments. The numbers at the top right corner indicate the timestamps (Min:Sec). Bar = 5 μm.

S1 Table

The nucleotides highlighted in red indicate the enzyme site for construction.

For knock out	
Mal3-KO-1F	GGTGGCGGCCGCTCTAGAAGATGTAAAGGTCGGCCATG
Mal3-KO-1R	CAAAAATGCTCCTTCAATCTAGACTTGATGGCAGCTCGTAAGC
Mal3-KO-3F	GGGTTCGCAAAGATAAAAGCTTGGCTGTAAAAATGCATTGGA
Mal3-KO-3R	GGTCGACGGTATCGATAAGCTTCCTCAGCGTATCATTCATGC
Mal3-text-1F	CAACGCACCTAAACTGACCA
Mal3-text-1R	GTTTGTCGACTTGGTGCTTG
Mal3-text-2F	GCTGACGTCTCCTAGGTTGT
Mal3-text-2R	GCTGATCTGACCAGTTGC
Mal3-text-3F	GTAACGCCTTCAGGCTCCGG
Mal3-text-3R	CTGCGTGAGCGCCATCTTAG
Mal3-ORF-F	ATGGGCGAATCGCGCCTATT
Mal3-ORF-R	GAACGTCTCCTGGTCGTCCA
For localization	
PBN-NaproMal3-mCherry-F	GCTATGACCATGATTACGAATTCGCTGACGTCTCCTAGGTTGT
PBN-NaproMal3-mCherry-R	CTTGCTCACCATGGATCCGGTACCGAACGTCTCCTGGTCGTCCA
PBN-NaproSLN1-mCherry-F	CCGAGCTCGGTACCGGATCCATGAGGATCGCCATCCGCGA
PBN-NaproSLN1-mCherry-R	CCCTTGCTCACCATGGATCCCGTCGCCACAGCAGCACCG
PBN-NaproSnc1-GFP-F	CGACGGCCAGTGCCAAGCTTATGCCCGAAGACGCTCCCTA
PBN-NaproSnc1-GFP-R	GCCCTTGCTCACCATCCCGGGGTTGCCCTTGAAGTGGAAAAC
PBN-NaproMal3-F	GCTATGACCATGATTACGAATTCGCTGACGTCTCCTAGGTTGT
PBN-NaproMal3-R	CTTGCTCACCATGGATCCGGTACCCCTCAGCGTATCATTCATGC
